# Effects of different anticoagulants on glycated albumin quantification

**DOI:** 10.11613/BM.2019.010901

**Published:** 2018-12-15

**Authors:** Graziella Bonetti, Nicola Di Gaetano, Renata Paleari, Ferruccio Ceriotti

**Affiliations:** 1 Central Clinical Chemistry Laboratory, ASST Spedali Civili, Brescia, Italy; 2 Instrumentation Laboratory - A Werfen Company, R&D Department, Milano, Italy; 3 Department of Pathophysiology and Transplantation, Centre for Metrological Traceability in Laboratory Medicine (CIRME), Università degli Studi di Milano, Milano, Italy; 4 Clinical Laboratory, Fondazione IRCCS Ca’ Granda Ospedale Maggiore Policlinico, Milano, Italy

**Keywords:** preanalytical phase, serum albumin, diabetes mellitus, anticoagulants

## Abstract

**Introduction:**

In the last 20 years glycated albumin (GA) measurement has been demonstrated to be a reliable glycation marker and recently as the most innovative one in western countries. Glycated albumin has been already adopted by some Asian countries due to its usefulness in diabetes screening. The aim of the present study was to investigate for the first time the effects of different anticoagulants on GA assay.

**Materials and methods:**

From each of 60 patients a serum tube and K_3_EDTA, Li-Heparin and NaF-EDTA containing tubes were collected. All tubes were from Sarstedt (Verona, Italy). Glycated albumin was measured in duplicate in each sample tube in a single analytical run with quantILab glycated albumin (Instrumentation Laboratory SpA - A Werfen Company, Milan, Italy) on Architect c8000 analyser (Abbott SRL, Rome, Italy). Comparison of GA% in evaluated tubes was made by paired Wilcoxon test.

**Results:**

Median and interquartile range GA% concentrations were 15.4% (13.2 - 19.1) in serum, 15.7% (13.6 - 19.9) in K_3_EDTA, 15.6% (13.3 - 19.7) in Li-heparin and 15.5% (13.1 - 19.3) in NaF-EDTA samples, respectively. Glycated albumin mean relative bias respect to serum was within desirable bias derived from biological variation studies (± 2.9%) when K_3_EDTA (+ 2.8%), Li-heparin (+ 0.9%) or NaF-EDTA (+ 0.1%), were used as anticoagulants.

**Conclusions:**

Our results demonstrate that the GA% assay is not affected by relevant interferences when K_3_EDTA, Li-heparin or NaF-EDTA are used as anticoagulants, so they can be used interchangeably without a relevant impact on the clinical use of the test.

## Introduction

Diabetes is the sixth of the deadliest diseases according to World Health Organization (WHO) and is considered the 21st century pandemic pathology for middle- and low-income countries (*1*). Fasting plasma glucose and glycated haemoglobin (HbA_1c_) are considered as gold standard for diabetes diagnosis and management (*2*). In the last 20 years glycated albumin (GA) evaluation has been demonstrated to be a reliable glycation marker and recently as the most innovative one in western countries. Glycated albumin is considered a good glycation marker because of albumin (Alb) abundance and localization and its high glycation speed, stated as 4.5-times higher than haemoglobin (*3*). Moreover, 15-day half-life of albumin makes GA capable of reflecting recent glucose exposure. Glycated albumin has been already adopted by some Asian countries due to its usefulness in diabetes screening (*4*). Recently, a study on GA usefulness in the diagnosis of diabetes in an Italian population has also been published (*5*). Glycated albumin is also useful for assessing glycaemic status in most of the clinical conditions where HbA_1c_ is less reliable such as anaemias and kidney impairment so that GA is currently indicated as the optimal marker in glycaemic control of diabetic nephropathy (*6*).

The study comes from the need of verifying the possibility to determine GA% in other materials, different from the one suggested by the manufacturer (serum), *i.e.* lithium-heparin (Li-Hep) plasma, used for the general chemistry or sodium fluoride (NaF) plasma used for glucose, or tripotassium-ethylenediaminetetraacetic acid (K_3_EDTA) obtained from the tubes used for cell blood count or HbA_1c_ determination. The aim of the present study was to investigate for the first time the effects of different anticoagulants on GA assay.

## Materials and methods

### Study design

This study was conducted in Central Clinical Chemistry Laboratory of Spedali Civili, Brescia, Italy, from 1^st^ to 30^th^ June 2016. We investigated leftover routine blood samples. The patients selected for this study had requests for HbA_1c_, glucose, clinical chemistry parameters and proteins analysis. No particular exclusion criteria were adopted. Since the biological materials used in this study were obtained from anonymized leftover routine specimens, informed consent from patients and ethical approval was unnecessary because patients were no longer traceable.

### Methods

Sixty patients were selected for a total of 240 samples (60 samples for each anticoagulant) to be analysed for GA concentration. From each patient four different S-Monovette® tubes were collected (Sarstedt, Verona, Italy): 1) serum 4.9 mL draw (ref. 04.1934), 2) Li-Hep, 4.9 mL draw (ref. 04.1936), 3) K_3_EDTA, 2.7 mL draw (ref. 04.1917.001) and 4) NaF-EDTA, 2.7 mL draw (ref. 04.1918).

Blood samples collected in NaF-EDTA, Li-Hep and no-additive containing tubes were centrifuged upon arrival in the laboratory at 2000xg for 15 min at room temperature using J6-MI centrifuge (Beckman Coulter, Milan, Italy). Blood samples collected in K_3_EDTA were centrifuged after HbA_1c_ determination (*i.e.* within 2-3 hours after blood drawing) at 2000xg for 15 min at room temperature using the same centrifuge. Serum and plasma samples were then aliquoted in 1.5 mL micro-tubes (ref.72.706 Sarstedt, Verona, Italy) and stored at - 20 °C until analysis.

Glycated albumin was determined in all serum and plasma samples with quantILab Glycated Albumin (Instrumentation Laboratory SpA - A Werfen Company, Milan, Italy) on Architect c8000 platform (Abbott Italia Abbott SRL, Rome, Italy), after calibration with ReferrIL Glycated Albumin (Instrumentation Laboratory SpA - A Werfen Company, Milan, Italy) (*7*).

The assay included separate measurements of GA (enzymatic method utilizing ketoamine oxidase and an albumin specific protease) and total albumin (bromocresol purple method) with the GA result expressed as a percentage of total albumin and corrected for adhering to high performance liquid chromatography (HPLC) results with an inter-method algorithm (*8*). Internal quality control SeraChem Glycated Albumin low and high (Instrumentation Laboratory SpA - A Werfen Company, Milan, Italy) were tested in all analytical runs according to the manufacturer. The different samples of the same subject were tested in duplicate, in a single analytical run, to reduce analytical variability.

### Statistical analysis

The statistical analysis was carried out with MedCalc for Windows, version 18.2.1 (MedCalc Software, Belgium). Bias from serum GA was calculated as: B = [ (GAval / GAser) x 100 ] – 100, where GAval represents GA% (mean of duplicate measurements) in each anticoagulant containing tube evaluated and GAser represents GA% (mean of duplicate measurements) in the serum tube. The maximal allowable bias was set at ± 2.9% according biological variation studies. The Kolmogorov-Smirnov test was used to assess the normality of distribution of investigated parameters. Not normally distributed data of different sample types were presented as median and interquartile range (IQR) and results obtained by different additive types were compared to serum results using the pairwise Wilcoxon test, where P < 0.05 was considered statistically significant.

## Results

**Figure 1:**
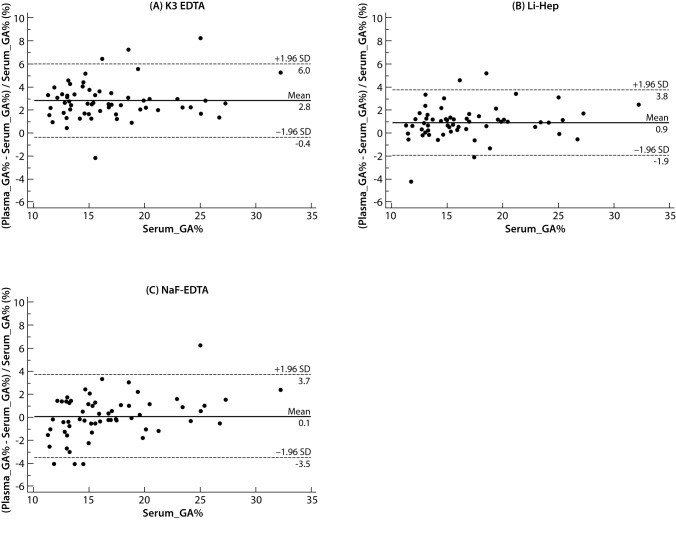
Bland-Altman plots showing the relative bias between GA% concentrations as measured in tripotassium-ethylenediaminetetraacetic acid (K_3_EDTA, A), lithium heparin (Li-Hep, B), sodium-fluoride ethylenediaminetetraacetic acid (NaF-EDTA, C) containing tubes and serum are presented. GA - glycated albumin. The solid line represents mean bias and dashed lines represent ± 1.96 SD interval.

Glycated albumin values in serum samples ranged from 11.3% to 32.2% covering both normal and abnormal values range. The results obtained in serum and other different matrix samples tested are summarized in Table 1 where data for all the measured parameters (*i.e.* GA%, GA and albumin) are reported. In presence of K_3_EDTA, the GA% values were found to be slightly increased with respect to those measured on serum (P < 0.001). Indeed, EDTA caused a negative bias in both GA and albumin assay, with a greater extent for albumin. As a result, the GA% values resulted slightly overestimated. For samples collected in Li-Hep, small differences in GA% values were seen respect to serum. No significant difference in GA% results were found between samples collected in NaF-EDTA and serum. In this case, the negative bias caused by NaF-EDTA in both GA and albumin quantification (- 15.1 and - 15.0%, respectively) was minimized when GA% was calculated. The Bland-Altman analysis for GA% measurements in plasma samples respect to serum are shown in Figure 1. A positive bias was systematically seen for samples collected in EDTA, except for only one. Mean absolute bias was 0.49 ± 0.36%. For samples collected in Li-Hep and NaF-EDTA the differences respect to serum were more limited, the absolute mean bias was 0.17 ± 0.26% and 0.05 ± 0.33%, respectively.

**Table 1:** Results obtained in serum and in plasma samples collected with different anticoagulants

**Tube**	**GA (%)** **(N = 60)**	**P**	**Mean bias (%)**	**GA (g/L)**	**P**	**Mean bias (%)**	**Albumin, g/L**	**P**	**Mean bias (%)**
Serum	15.4 (13.2-19.1)	-	-	6.1 (5.1-7.5)	-	-	43.0 (39.8-45.3)	-	-
K_3_EDTA	15.7 (13.6-19.9)	< 0.001	+ 2.8	6.0 (5.0-7.5)	< 0.001	- 1.9	40.1 (38.0-43.0)	< 0.001	- 5.1
Li-Hep	15.6 (13.3-19.7)	< 0.001	+ 0.9	6.1 (5.1-7.5)	0.281	+ 0.4	42.2 (39.7-45.4)	0.022	- 0.7
NaF-EDTA	15.5 (13.1-19.3)	0.403	+ 0.1	5.0 (4.2-6.6)	< 0.001	- 15.1	35.5 (34.1-38.6)	< 0.001	- 15.0
Data are expressed as median and interquartile range (in brackets). Differences between plasma and serum were tested using paired Wilcoxon test. The mean relative bias respect to the results obtained in serum is also presented. P < 0.05 was considered statistically significant. K_3_EDTA - tripotassium-ethylenediaminetetraacetic acid. Li-Hep - lithium heparin. NaF - sodium-fluoride. GA - glycated albumin.									

## Discussion

The present study is the first one evaluating the effect of different anticoagulants on GA%. Although there is a statistically significant difference between GA% values observed in samples collected in K_3_EDTA and Li-Hep with respect to serum, it is not clinically relevant because it does not exceed the desirable bias quality specification of ± 2.9% based on GA biological variation data (*9*). Some misaligned results would be attributed to known EDTA lowering effects on albumin concentration that were more evident in the albumin assay than in GA assay (*10*). The number of evaluated samples represents a limitation for this study; a larger dataset could provide a more robust view of the obtained results.

In conclusion, our results demonstrate that the GA% assay is not affected by relevant interferences when K_3_EDTA, Li-Hep or NaF-EDTA are used as anticoagulants; so they can be used interchangeably for sample collection without any relevant impact on the clinical use of the test.
